# Do Neighborhoods Make People Active, or Do People Make Active Neighborhoods? Evidence from a Planned Community in Austin, Texas

**DOI:** 10.5888/pcd10.120119

**Published:** 2013-06-20

**Authors:** Tamara Vehige Calise, Timothy Heeren, William DeJong, Samuel C. Dumith, Harold W. Kohl

**Affiliations:** Author Affiliations: Timothy Heeren, Boston University School of Public Health, Department of Biostatistics, Boston, Massachusetts; William DeJong, Boston University School of Public Health, Department of Community Health Sciences, Boston, Massachusetts; Samuel C. Dumith, Federal University of Pelotas, Pelotas, Brazil; Harold W. Kohl, III, University of Texas Health Science Center – Houston, School of Public Health, Houston, Texas, Michael and Susan Dell Center for Healthy Living and University of Texas, Austin, Texas.

## Abstract

**Introduction:**

Whether patterns of physical activity in different communities can be attributed to the built environment or instead reflect self-selection is not well understood. The objective of this study was to examine neighborhood preferences and behavior-specific physical activity among residents who moved to a new urbanist-designed community.

**Methods:**

We used data from a 2009 survey (n = 424) that was designed and administered to evaluate neighborhood preferences and behavior-specific physical activity before and after residents moved. Data were grouped and stratified by pre-move physical activity levels into low-, middle-, and high-activity groups. We used Student’s paired sample *t* test and Wilcoxon signed-rank test to compare pre- and post-move scores and used an analysis of variance to compare mean changes as a function of pre-move physical activity level.

**Results:**

After moving, the high-activity group continued to be significantly more active than the middle- and low-activity groups (*P* < .001). However, we saw the biggest increase in pre- to post-move total physical activity in the low-activity group (mean increase, 176.3 min/wk) compared with the middle- (mean increase, 69.5 min/wk) and high-activity groups (mean decrease, 67.9 min/wk). All 3 groups had significant increases in walking inside the neighborhood for recreation. The preferred neighborhood features with the most significant pre- to post-move change scores were those associated with greater walkability.

**Conclusion:**

This study supports the role the environment plays in physical activity. These data suggest that moving to an activity-friendly neighborhood can positively affect physical activity levels, particularly among residents who had previously been least active.

## Introduction

Research has shown that the environment influences physical activity (1). Compared with residents of vehicle-centered developments, residents of neighborhoods guided by new urbanist principles (2) are more likely to be physically active. Such neighborhoods are characterized by environmental supports including recreational facilities, high-density, mixed land use, and connected street networks (1,3–5).

Whether patterns of physical activity in different neighborhoods can be attributed to the built environment or whether, instead, patterns reflect the self-selection of residents into an environment that support their values is not well understood. A small but growing body of literature has examined these relationships. Most studies have been cross-sectional and have compared behaviors of residents living in neighborhoods with varying levels of walkability (6–9). The focus has typically been on transport-related behaviors rather than physical activity for recreation and transport (3,10–22). Researchers used advanced statistics to control for preferences (10–12,15–18,20–24) rather than a research design that could help rule out alternative explanations for changes in physical activity.

Information on residents’ preferences and behavior-specific physical activity before and after moving to neighborhoods is needed to better understand self-selection and its association to the environment and physical activity. Such information could guide policy and the development or redevelopment of neighborhoods. However, the implausibility of randomly assigning people to live in different neighborhoods makes research difficult.

The purpose of our study was to examine neighborhood preferences and behavior-specific physical activity changes among residents who moved to a new urbanist-designed housing development.

## Methods

We used a 150-question cross-sectional survey (Appendix) to assess neighborhood preferences and physical activity levels before and after residents moved to Mueller, a new urbanist-designed development in Austin, Texas. This study design treats study participants as their own controls.

At the time of data collection (May – August 2009), 324 acres of the 700-acre development had been completed, including 424 homes, 400,000 square feet of retail space, 890,000 square feet of commercial and office space, and 70 acres of the 140 acres of planned parks and open spaces, including 5 miles of trails. The institutional review boards for the Boston University Medical Center and University of Texas Health Science Center – Houston approved the study.

We included all homes with electricity that had been activated before April 1, 2009, in our study sample (n = 424). By mail, we invited 1 adult aged 18 years or older from each home who understood English and was free from physical impairments that limited his or her ability to walk to complete a survey. Nonresponders received several mailed reminders. Respondents who completed and returned the survey received a $15 grocery store gift card. Three surveys were undeliverable or ineligible for inclusion, leaving 421 eligible households. In all, residents returned 267 surveys (63.4% response rate). Approximately 70.0% of the surveys were returned before July 2009.

Survey items included demographics, addresses, length of residence in previous neighborhood, length of residence in Mueller, desired neighborhood characteristics, and physical activity behaviors. We used addresses to assess population density of respondents’ previous neighborhoods and of Mueller on the basis of US Census Bureau American Community Survey 2005–2009 Estimates block group data (25).

### Measures

We used the Neighborhood Physical Activity Questionnaire (NPAQ) (26) to assess recreational and transport-related walking and biking undertaken both within and outside the neighborhood, plus other moderate- and vigorous-intensity physical activity. The NPAQ was based on the validated International Physical Activity Questionnaire (27) and has been found to be reliable (26). A measure of duration reported as minutes per week was used for each variable.

We created measures of total activity within each domain: 1) total transport-related physical activity is the sum of the times spent walking and biking for transport, both inside and outside the neighborhood; 2) total recreational walking and biking is the sum of the times spent walking and biking for recreation, both inside and outside the neighborhood; 3) total recreational physical activity is the sum of the reported times spent walking and biking for recreation, both inside and outside the neighborhood, plus reported moderate- and vigorous-intensity physical activity time; and 4) total physical activity is the sum of the times spent walking and biking plus engaging in other moderate- and vigorous-intensity activities.

Using a 5-item Likert scale (1, not at all important; 2; 3, somewhat important; 4; and 5, very important), respondents reported the importance of 13 neighborhood characteristics in their decision to move to their previous neighborhood and then, in a later section of the survey, their decision to move to Mueller. The listed characteristics (eg, closeness to job, shops, recreational facilities; ease of walking) are consistent with those examined in previous studies (28,29) and were chosen on the basis of their potential effect on physical activity (5,30).

### Analyses

All statistical analyses were conducted using STATA SE/11.0 (StataCorp, College Station, Texas); α levels were set at 0.05. A survey participant's records were excluded if any component data were missing. Fisher’s exact tests were used to test associations between categorical variables. *P*-values present in tables and text are not adjusted for multiple comparisons; however, results of Hochberg’s adjustment for multiple comparisons are footnoted in the tables. Pre- to post-move differences were calculated for neighborhood preferences by subtracting the pre-move score from the post-move score. We used both the paired sample *t* test and Wilcoxon signed rank test to calculate statistical tests comparing the pre- and post-move scores for each item.

Tertiles of pre-move total physical activity scores were used to stratify respondents as low (0–180 min), middle (181–420 min), or high (≥421 min). For each measure of physical activity, we used 1-way analysis of variance (ANOVA) to compare mean changes in pre- to post-move physical activity as a function of pre-move physical activity level. An ANOVA was also used to compare the mean level of importance of neighborhood characteristics in respondents’ decision to move to Mueller as a function of changes in pre-move physical activity.

## Results

Approximately 62.0% of the respondents were female, 88.6% were white, 93.2% had a college or postgraduate degree, 88.6% were employed, and 54.1% were aged 20 to 39 years. Roughly 45% reported an annual household income greater than $90,000. Respondents reported a mean of 10.1 months’ residence in Mueller and 50.2 months in their previous home.

The demographics did not differ significantly among the low-, middle-, and high-activity groups. Approximately 67% of the low-activity group were female compared with 53% of the middle- and 65% of the high-activity groups; 96% of the low-activity group had a college or postgraduate degree compared with 90.0% of the middle- and 95.0% of the high-activity groups; 57.0% of the low- and middle- activity groups were aged 20 to 39 years compared with 48.0% of the high-activity group; and 36.0% of the low-activity group reported an annual household income greater than $90,000 compared with 48% of the middle- and 46% of the high-activity groups. Thirty-eight percent of the low-activity group had lived in Mueller for 12 to 20 months compared with 36% of the middle- and 45% of the high-activity groups.

Almost 87.0% of the respondents (n = 231) had moved to Mueller from another area of Austin. Approximately 25.0% had moved from neighborhoods with fewer than 881 people per square kilometer, and 25.0% had moved from neighborhoods with greater than 2,223 people per square kilometer. Mueller had a population density below 881 people per square kilometer at the time of our study.

Results of both the paired sample *t* test and Wilcoxon signed-rank test were the same for all but 2 of the preference items. The paired sample *t* tests showed significant pre- to post-move changes in mean scores for most of the neighborhood preference dimensions (Table 1). In summary, the preferred neighborhood features with the most significant pre- to post-move change scores were those associated with greater walkability (ie, being close to open space and parks, recreational facilities, shops, services, and restaurants). Several reasons were not significantly different between neighborhoods: affordability/value, access to freeways, and quality of schools, although the latter variable was significant according to the Wilcoxon test, *P* = .02. Additionally, safety from crime was significant according to the paired *t* test (*P* = .04), it was not significant according to the Wilcoxon test (*P* = .12).

**Table 1:** Importance of Neighborhood Characteristics in Residents’ (N = 266) Decision to Move, Previous Neighborhood vs Mueller Urban Development, Austin, Texas, 2009 a

Characteristic	Previous Neighborhood, Mean (95% CI)	Mueller, Mean, (95% CI)	Change, Mean (95% CI)	*P* Value^b^
Affordability/value	4.2 (4.0– 4.3)	4.2 (4.1– 4.3)	0.0 (−0.1 to 0.2)	.58
Closeness to open space such as parks	3.0 (2.9–3.2)	4.4 (4.3–4.5)	1.3 (1.2–1.5)	<.001
Closeness to job or school	3.7 (3.5–3.8)	3.9 (3.8–4.1)	0.2 (0.1–0.4)	.01
Closeness to public transportation	2.1 (1.9–2.2)	2.8 (2.7–3.0)	0.8 (0.6–0.9)	<.001
Closeness to shops, services, and restaurants	3.3 (3.2–3.5)	4.0 (3.9–4.1)	0.7 (0.5–0.9)	<.001
Ease of walking	3.0 (2.8–3.1)	4.2 (4.0–4.3)	1.2 (1.0–1.4)	<.001
Sense of community	3.0 (2.9–3.2)	4.2 (4.1–4.3)	1.2 (1.0–1.3)	<.001
Safety from crime	3.6 (3.5–3.8)	3.8 (3.6–3.9)	0.2 (0.0–0.3)	.04^c^
Quality of schools	2.2 (2.1–2.4)	2.3 (2.1–2.4)	0.1 (−0.1 to 0.3)	.39^d^
Closeness to recreational facilities	2.8 (2.7–3.0)	3.5 (3.3–3.6)	0.7 (0.5–0.9)	<.001
Access to freeways	3.2 (3.0–3.3)	3.3 (3.1–3.4)	0.1 (−0.0–0.2)	.15
Closeness to health care facilities	2.3 (2.2–2.5)	2.7 (2.5–2.8)	0.4 (0.2–0.5)	<.001
Closeness to cultural and entertainment choices	3.2 (3.0–3.3)	3.8 (3.6–3.9)	0.6 (0.4–0.8)	<.001

Abbreviation: CI, confidence interval.

a Mean scores are based on a 5-pt Likert Scale (1, not at all important; 2; 3, somewhat important; 4; and 5, very important).

b *P*-values based on paired sample *t* tests. *P* values of .01 or less remain significant after controlling for multiple comparisons via Hochberg’s method.

c Variable not significant according to Wilcoxon signed-rank test, *P* = .12.

d Variable significant according to Wilcoxon signed-rank test, *P* = .02.

Overall, after moving to Mueller, the high-activity group continued to be significantly more active than the middle- and low-activity groups (*P* < .001) (high-activity group, mean 637.3 min total physical activity/wk; middle-activity group, mean 360.2 min total physical activity/wk; and low-activity group, mean 258.1 min total physical activity/wk). For all 3 groups, the majority of post-move physical activity was recreational (*P* < .001) (high-activity group, mean 555.4 min total recreational physical activity/wk; middle-activity group, mean 339.5 min total recreational physical activity/wk; and low-activity group, mean 251.8 min total recreational physical activity/wk).

Although the high-activity group stayed the most active post-move, the low- and middle-activity groups had an increase in reported total physical activity (mean 176.3 min/wk and 69.5 min/wk, respectively), whereas physical activity in the high-activity group declined by an average of 67.9 minutes per week (*P* < .001). Similarly, the low- and middle-activity groups had a reported mean increase of total recreational activity per week (178.8 min/wk and 77.4 min/wk, respectively), and the high-activity group had a reported mean decrease of 44.5 minutes per week (*P* < .001). The low-activity group was the only group to report greater post-move transport-related activity with an average increase of 14.3 minutes per week, compared with the middle- and high-activity groups (mean decrease 6.3 min/wk and 22.0 min/wk, respectively; *P* = .03).

We collected behavior-specific information on respondents (Table 2). All 3 groups reported substantial increases in recreational walking inside the neighborhood (mean increase 100.7 min/wk, low-activity group; 47.3 min/wk, middle-activity group; 56.5 min/wk, high-activity group; *P* = .05). Walking for recreation outside the neighborhood decreased (mean decrease 2.0 min/wk, low-activity group; 18.2 min/wk, middle-activity group; 40.2 min/wk high-activity group; *P* = <.001). The low-activity group did not change its walking behaviors related to transport inside the neighborhood from pre- to post- move (0.8 min/wk), whereas the middle- and high-activity groups both reported a decrease (mean 7.9 min/wk, middle-activity group; 20.3 min/wk, high-activity group, *P* =.10).

**Table 2:** Changes in Mean Minutes of Physical Activity Per Week Stratified by Pre-Move Physical Activity Levels, Mueller Urban Development, Austin, Texas, 2009

Physical Activity Domain	Low-Activity Group^a^ (n = 92), Mean (SD)	Middle-Activity Group^b^, (n = 86), Mean (SD)	High-Activity Group^c^, (n = 81), Mean (SD)	*P* Value^d^
**Recreational/leisure physical activity^e^**				
Moderate-intensity	24.1 (80.7)	18.3 (95.4)	3.7 (165.9)	.52
Vigorous-intensity	40.1 (109.3)	16.0 (75.0)	−27.5 (142.3)	<.001
Total moderate and vigorous	64.6^f^ (140.9)	34.3 (139.2)	−17.9^f^ (231.7)	.009
**Recreational walking**				
Inside neighborhood	100.7 (143.2)	47.3 (125.6)	56.5 (190.0)	.05
Outside neighborhood	−2.0 (27.6)	−18.2 (60.2)	−40.2 (79.1)	<.001
Total recreational walking	98.7 (146.8)	29.7^f^ (133.7)	3.5^f^ (180.5)	<.001
**Recreational biking**				
Inside neighborhood	14.1 (33.4)	7.2 (53.9)	−9.9 (110.9)	.09
Outside neighborhood	2.2 (12.3)	8.6 (38.6)	−22.5 (83.0)	<.001
Total recreational biking	16.3 (38.8)	15.9^f^ (68.0)	−32.6^f^ (168.2)	.003
**Total recreational walking and biking**				
Walking and biking in neighborhood	115.3 (145.5)	53.6 (144.7)	46.5 (216.5)	.01
Walking and biking outside neighborhood	0.2 (30.4)	−9.1 (79.2)	−63.3 (120.6)	<.001
Total recreational walking and biking	115.5 (152.6)	45.1^f^ (171.0)	−29.9^f^ (232.9)	<.001
**Total recreational physical activity**				
Moderate-intensity	137.8 (174.6)	62.4 (194.5)	−16.0 (288.0)	<.001
Vigorous-intensity	40.1 (109.3)	16.0 (75.0)	−27.5 (142.3)	<.001
Total moderate and vigorous	178.8^f^ (220.5)	77.4^f^ (219.3)	−44.5^f^ (331.1)	<.001
**Walking for transport**				
Inside neighborhood	0.8 (35.5)	−7.9 (45.4)	−20.3 (97.6)	.10
Outside neighborhood	4.1 (14.1)	−1.6 (15.3)	−4.2 (48.3)	.17
Total transport walking	3.8^f^ (40.5)	−10.0^f^ (48.0)	−24.6^f^ (109.1)	.04
**Biking for transport**				
Inside neighborhood	6.0 (22.3)	3.8 (22.3)	1.3 (57.8)	.73
Outside neighborhood	4.1 (26.1)	1.9 (17.3)	1.2 (42.9)	.80
Total transport biking	10.2^f^ (38.3)	5.2^f^ (36.4)	2.5 (80.9)	.65
**Total transport physical activity**				
Walking and biking in neighborhood	6.9 (44.8)	−5.7 (50.1)	−18.9 (112.3)	.08
Walking and biking outside neighborhood	8.3 (29.5)	0.3 (23.5)	−3.1 (67.3)	.22
Total transport walking and biking	14.3^f^ (60.8)	−6.3^f^ (55.8)	−22.0 (133.4)	.03
**Total physical activity^g^**				
Moderate-intensity^h^	140.1 (159.7)	52.7 (202.7)	−39.2 (289.4)	<.001
Vigorous-intensity	40.1 (109.3)	16.0 (75.0)	−27.5 (142.3)	<.001
Moderate- and vigorous-intensity^h^	176.3^f^ (188.1)	69.5^f^ (227.4)	−67.9^f^ (321.7)	<.001

Abbreviation: SD, standard deviation.

a People in the low pre-move total physical activity group engaged in 0–180 min/wk.

b People in the middle pre-move total physical activity group engaged in 181–420 min/wk.

c People in the high pre-move total physical activity group engaged in ≥ 421 min/wk.

d *P*-values based on one-way ANOVAs. *P*-values of .003 or less remain significant after controlling for multiple comparisons via Hochberg’s method.

e Recreational physical activity excluding outdoor walking and biking.

f Individual means do not sum to total means because of missing data.

g All domains combined: moderate intensity = recreational/leisure-time moderate intensity and walking/biking for recreation and transport; moderate and vigorous intensity = moderate intensity + vigorous intensity.

h Moderate intensity = recreational moderate intensity + walking and biking; total moderate and vigorous intensity = moderate intensity + vigorous intensity.

We saw the biggest increases in physical activity in the low-activity group, followed by the middle-activity group. However, when considering the importance of Mueller’s characteristics stratified by changes in pre-move physical activity (Table 3), the high-activity group reported a significantly higher level of importance on closeness to open space and parks on the Likert scale (4.60) than the low- (4.22) and middle-activity (4.36) groups (*P* = .02). Although not significant, the high-activity group also reported a higher level of importance on ease of walking (4.32 Likert scale [1, not at all important; 2 and 3, somewhat important; 4 and 5, very important]) compared with 4.13 (low-activity group) and 4.09, middle-activity group (*P* = .24) and closeness to recreational facilities (3.71, high-activity group; 3.43 low-activity group; 3.33, middle-activity group; *P* = .11).

**Table 3:** Importance of Mueller Neighborhood Characteristics Stratified by Changes in Pre-Move Physical Activity Levels, Mueller Urban Development, Austin, Texas, 2009.

Neighborhood Characteristics^a^	Low-Activity Group^b^, (n = 92), Mean (+SD)	Middle-Activity Group^c^, (n = 86), Mean (+SD)	High-Activity Group^d^, (n = 81), Mean (SD)	*P* Value^e^
Affordability/value	4.22 (0.89)	4.03 (1.16)	4.36 (0.86)	.09
Closeness to open space such as parks	4.22 (1.02)	4.36 (0.95)	4.60 (0.68)	.02
Closeness to job or school	3.8 (1.33)	3.92 (1.16)	4.01 (1.23)	.62
Closeness to public transportation	2.88 (1.31)	2.84 (1.27)	2.77 (1.32)	.86
Closeness to shops, services, and restaurants	4.09 (0.90)	3.8 (1.01)	4.02 (0.91)	.23
Ease of walking	4.13 (0.96))	4.09 (0.98)	4.32 (0.86)	.24
Sense of community	4.17 (1.02)	4.10 (0.97)	4.27 (0.81)	.51
Safety from crime	3.82 (0.98)	3.58 (1.07)	3.88 (1.04)	.13
Quality of schools	2.28 (1.20)	2.12 (1.04)	2.46 (1.37)	.21
Closeness to recreational facilities	3.43 (1.21)	3.33 (1.27)	3.71 (1.06)	.11
Access to freeways	3.40 (1.26)	3.08 (1.20)	3.27 (1.22)	.22
Closeness to health care facilities	2.85 (1.28)	2.53 (1.21)	2.68 (1.35)	.24
Closeness to cultural and entertainment choices	3.73 (1.14)	3.80 (1.03)	3.74 (1.11)	.90

a Mean scores for neighborhood characteristics are based on a 5-pt Likert Scale (1, not at all important; 2; 3, somewhat important; 4; and 5, very important).

b People in the low pre-move total physical activity group engaged in 0–180 min/wk.

c People in the middle pre-move total physical activity group engaged in 181–420 min/wk.

d People in the high pre-move total physical activity group engaged in ≥ 421 min/wk.

e *P*-values based on one-way ANOVAs. None of these *P* values remain significant after controlling for multiple comparisons via Hochberg’s method.

## Discussion

A major challenge to the validity of studies on the effect of environment on physical activity is determining whether residents with a predisposition to be active choose a neighborhood that is more supportive of physical activities (1). This study was among the first to investigate residential preferences and physical activity behaviors.

The neighborhood features associated with the most significant change scores were those associated with greater walkability, such as closeness to open spaces, parks, recreational facilities, and shops; ease of walking; and sense of community. Similarly, Mumford et al (31) found that residents who moved to a development similar to Mueller, in Atlanta, Georgia, selected the neighborhood for reasons associated with walkability. Giles-Corti et al (29) also found that more than half of respondents rated aspects of neighborhood walkability as important factors in their decisions to move to a new location.

Although respondents may have decided to move to Mueller partly on the basis of their desired level of physical activity, the environment itself also seemed to play a role in their decision. The biggest increase in physical activity was seen among those who were the least active before moving to Mueller. More specifically, the low-activity group had the most significant increase in walking and biking for recreation inside the neighborhood and was the only group to increase transport-related physical activity after moving to Mueller. However, the high-activity group reported attaching a higher level of importance to neighborhood characteristics that are supportive of these behaviors.

Although Mueller’s master plan calls for a town center with restaurants and shops, the closest amenities were between one-half and 1 mile away from the developed houses at the time of data collection. Access to and from Mueller was also limited because the surrounding arterial roadways were not pedestrian-friendly. Although respondents may have considered closeness to restaurants and shops to be important, the data suggest that the limited amenities within close distance may explain the low level of walking and biking for transport among all groups.

Several limitations of this study should be noted. This was a cross-sectional study that retrospectively measured self-reported pre-and post-move neighborhood preferences and physical activity. However, because individuals were used as their own controls, confounding lifestyle variables were less of a problem than in cross-sectional studies comparing individuals living in different neighborhoods. Moreover, it is not feasible to randomly assign people to live in different neighborhoods. Our design allowed for the identification of specific behavioral changes and neighborhood preferences pre- to post-move.

Self-report measures have well-known challenges, including the unknown validity of the NPAQ. However, NPAQ was based on the validated IPAQ, and the results are systematically reliable across subjects; therefore, there should not be intergroup measurement errors. The use of recall-based, pre-move data is also a limitation (32,33). Residents had lived in Mueller an average of 10 months at the time of our study, and, therefore, we cannot rule out the possibility that recollection of previous neighborhood preferences and behaviors may be inaccurate. This problem may be particularly evident among respondents who had lived in Mueller for longer periods of time. However, we did not find significant differences in physical activity among those who had lived in Mueller for less than 6 months, for 6 to 11 months, or for more than 1 year. Moreover, the subjective self-report data found in this study are consistent with objective accelerometer values found in a study that compared high- and low-walkability neighborhoods (7). Given the challenges to conducting longitudinal studies, more research is needed on the validity of these retrospective self-reports.

Overall, our study reported high levels of physical activity. This finding could be explained in several ways. First, respondents may have chosen to move to Mueller because it supports their desire to be active. The high-activity group did report attaching a significantly higher level of importance on closeness to open space and parks and a higher level of importance on ease of walking and closeness to recreational facilities than the low- and middle-activity groups. However, the low- and middle-activity groups had an increase in reported total physical activity unlike the high-activity group, which suggests that the environment itself, rather than personal preferences, could have had an effect. Another explanation for the high levels of physical activity is that 1 adult from each household was asked to complete the survey. Seeing that the survey concerned physical activity, the more active adult in the household may have opted to respond. Regardless, there were substantial increases in activity within the low-activity group.

Respondents may have thought it was socially desirable to represent their current physical activity more favorably. Although we cannot rule this out, we did not see behavior-specific increases across the board nor did we see increases in total physical activity for the high-activity group. The high levels of activity might also reflect a response bias, namely, that households with active adults may be more likely to return surveys than households with inactive adults. Although the influence of the 36% nonresponders is unknown, a 63.4% response rate suggests our results are generalizable to Mueller. Although the demographic characteristics of Mueller residents are similar to those of residents living in other new urbanist-designed communities (29,31,34), the generalizability is unknown.

Regression to the mean could partially explain the greater increases in physical activity among the low-activity group and the decreases among the high-activity group. Studies that measure change are frequently at risk of being affected by regression to the mean. However, this study found an overall reported increase in physical activity per week after residents moved to Mueller (66.4 min; 95% CI [32.8, 100.1], results fully described elsewhere) (35). Thus, regression to the mean cannot fully explain the observed changes in physical activity for several reasons. First, there were significant increases in physical activity of the middle-activity group. If regression to the mean were fully operating, we would not see these increased levels of physical activity. Second, the decrease in physical activity reported by the high-activity group was only about one-third the magnitude of the increase reported by the low-activity group. If the observed changes were only a result of the regression to the mean, the increase within the low-activity group would have been about the same as the decrease in the high-activity group.

Notwithstanding these limitations, this study appears to be 1 of the few to examine both pre- and post-move preferences in neighborhood features and physical activity. It helps to advance the research toward a better understanding of efforts to alter environments to promote population shifts in physical activity. Public health professionals can use data such as these to execute environmental changes that are supportive of physical activity and to justify relevant policies to work with departments of planning, transportation, public works, and economic growth and development. Our study data provide some support for the possible role the environment plays in physical activity. Future research should feature prospective study designs, investigate individual neighborhood characteristics that promote greater physical activity among new residents, and include more diverse samples.
